# Exploring the role of attention towards balance in chronic dizziness: Development of the Balance Vigilance Questionnaire

**DOI:** 10.1111/ene.16148

**Published:** 2023-11-28

**Authors:** Toby J. Ellmers, Elmar C. Kal

**Affiliations:** ^1^ Centre for Vestibular Neurology, Department of Brain Sciences Imperial College London London UK; ^2^ Centre for Cognitive Neuroscience, Department of Health Sciences, College of Health, Medicine, and Life Sciences Brunel University London Uxbridge UK

**Keywords:** attention, dizziness, functional disorders, hypervigilance, PPPD, vestibular

## Abstract

**Background and purpose:**

Vigilance towards balance has been proposed to underpin various chronic dizziness disorders, including persistent postural–perceptual dizziness (PPPD). The objective of this study was to develop (through patient input) a validated balance‐specific measure of vigilance that comprehensively assesses the varied ways in which this construct may manifest.

**Methods:**

We developed the Balance Vigilance Questionnaire (Balance‐VQ) through patient and clinician feedback, designed to assess vigilance towards balance. We then validated the questionnaire in 497 participants consisting of patients diagnosed with chronic dizziness disorders (including 97 individuals diagnosed with PPPD) and healthy controls.

**Results:**

The final six‐item Balance‐VQ was shown to be a valid and reliable way to assess vigilance towards balance. Scores were significantly higher in individuals diagnosed with PPPD compared to controls. Although scores were also higher in the PPPD group compared to individuals with diagnosed vestibular disorders other than PPPD, Balance‐VQ scores did not discriminate between the two groups when confounding factors (including dizziness severity) were controlled for. Scores did, however, independently discriminate between the PPPD group and individuals who experience dizziness in daily life, but who have not been diagnosed with a neuro‐otological disorder.

**Conclusions:**

Our findings confirm that the Balance‐VQ is a valid and reliable instrument for assessing vigilance towards balance. As symptom vigilance has been identified as a key risk factor for developing chronic dizziness following acute vestibular symptoms or balance disruption, we recommend using the Balance‐VQ as a screening tool in people presenting with such symptoms.

## INTRODUCTION

Vigilant monitoring of body signals has been associated with the development and maintenance of a range of clinical balance disorders, including functional gait disorders [1, 2, 3, 4] and disorders of chronic dizziness, such as persistent postural–perceptual dizziness (PPPD) [5, 6, 7, 8, 9]. PPPD is a recently defined disorder of chronic dizziness, characterized by nonspinning vertigo, perceived/subjective unsteadiness, and hypersensitivity to motion [5]. Symptoms associated with PPPD often develop following acute vestibular symptoms or balance disruption [5, 6, 7]. Although some degree of vigilance towards balance likely reflects an adaptive response to vestibular dysfunction and imbalance, a recent systematic review concluded that continuous monitoring of balance appears to be a strong risk factor for developing persistent dizziness symptoms [8].

Although the specific mechanisms through which balance vigilance may contribute to dizziness remain unknown, some researchers have hypothesized that heightened monitoring of balance in PPPD leads to greater awareness of minor (otherwise subconscious) discrepancies between anticipated and actual postural feedback signals [6, 7]. This mismatch may then lead to a distorted sense of imbalance and feelings of dizziness. This idea is supported by our recent experimental work highlighting how balance vigilance contributes to the acute formation of distorted perceptions of unsteadiness in healthy (older) adults without vestibular deficits [10]. Balance vigilance has also been proposed to play a role in the maintenance of "unexplained dizziness" in older adults [10]. Here, dizziness is characterized by vague—and distorted—feelings of unsteadiness and imbalance, despite a lack of readily identifiable neuro‐otological dysfunction [11, 12].

Comprehensive understanding of the specific role that balance vigilance plays in the maintenance of clinical balance disorders is therefore important for developing future therapeutic strategies. However, this requires a uniformly used and validated tool to specifically assesses balance vigilance. Although a Body Vigilance Scale [13] exists, this tool was developed to assess generalized vigilance in panic disorder, as well as the amount of attention directed towards monitoring a broad range of sensations not inherently related to balance (e.g., upset stomach, heart palpitations, shortness of breath). Although one item (out of 18) does ask about the amount of attention directed towards feelings of dizziness, this scale is not validated for separation of individual items. Furthermore, restricting assessment of balance vigilance to a single item hinders our ability to perform a fine‐grained exploration of this construct. As the broad items contained in this scale fail to distinguish between healthy controls and individuals with balance disorders, such as PPPD [14], there is a need to develop (through patient input) a validated balance‐specific measure of vigilance that provides a comprehensive yet efficient assessment of the varied ways in which this construct may manifest. Doing so will also help identify those most likely to benefit from specific, tailored therapeutic strategies. This is the aim of the present work.

## METHODS

### Participants

A total of 555 participants (including healthy controls and individuals experiencing chronic and acute dizziness) were recruited from social support groups for older adults and patient support networks for people living with balance problems or dizziness within the UK, North America, and Australia. The survey was disseminated via social media posts, as well as newsletters sent to group members, and no incentives were provided for participation. Unfortunately, due to these recruitment methods used, it was not possible to determine the response rate. Although individuals with PPPD were our primary focus, we were also interested in exploring the role of balance vigilance in older adults experiencing chronic dizziness. Due to the incidence peak of chronic dizziness at middle‐age and older [14, 15], we therefore restricted participation to those aged 30 years and older, to prevent collecting a much younger control group of "non‐dizzy" individuals (e.g., avoiding a non‐dizzy control group of only college‐aged students). Participants were excluded if they had been diagnosed with dementia or any other degenerative neurological disease. The final sample consisted of 97 individuals diagnosed with PPPD, 97 with a diagnosed vestibular disorder other than PPPD, and 303 controls without diagnosed neuro‐otological dysfunction. (However, 98 of 303 did report experiencing some degree of “dizziness that is unrelated to alcohol or drug consumption, or standing up too quickly.” We thus also separated control participants into "dizzy" and "non‐dizzy" groups for certain analyses; see the Statistical Analysis section for further information.) Please see Table 1 for a full demographic breakdown of the sample. Ethical approval was obtained from the research ethics committee of the College of Health, Medicine, and Life Sciences of Brunel University London (REF# 24087), and the research was carried out in accordance with the Declaration of Helsinki. All participants provided written informed consent.

**TABLE 1:** Participant characteristics.

Characteristic	Vestibular, *n* = 97	PPPD, *n* = 97	Controls, no neuro‐otological dysfunction, *n* = 303	
			Control–dizzy, *n* = 98	Control–non‐dizzy, *n* = 203^a^
Background/general health				
Age, years, mean ± SD [range]	62.8 ± 13.6 [31–87]	47.2 ± 11.2 [30–76]^b^	70.9 ± 10.3 [34–90]^a^	68.2 ± 15.5 [30–90]
Female gender, *n* (%)	86 (91%)^b^	79 (82%)^a^	80 (83%)^a^	144 (71%)
Education level: college/sixth form or above, *n* (%)	87 (90%)^a^	79 (82%)^a^	81 (85%)^b^	179 (89%)^b^
General health: self‐reporting very good to excellent health, *n* (%)	31 (32%)^a^	19 (20%)	52 (53%)	126 (63%)^b^
Medication use > 4, *n* (%)	14 (15%)^a^	8 (8%)	16 (16%)	27 (13%)
Peripheral neuropathy, *n* (%)	3 (3%)^a^	2 (2%)	10 (10%)^a^	6 (3%)^b^
Diabetes, *n* (%)	5 (5%)^c^	6 (6%)^a^	9 (9%)	20 (10%)^c^
Physical functioning				
Falls in past 12 months, *n* (%)	41 (43%)^a^	31 (32%)^a^	43 (44%)^a^	53 (26%)^b^
Balance problems, *n* (%)	74 (79%)^c^	84 (87%)	50 (51%)	58 (29%)^e^
Walking aid, *n* (%)	12 (13%)^c^	11 (11%)	14 (14%)^a^	14 (7%)^b^
ADL assistance, *n* (%)	4 (4%)^b^	8 (8%)	1 (1%)^a^	0 (0%)^d^
Psychological functioning				
Short Falls Efficacy Scale–International, 7–28, mean ± SD [range]	12.0 ± 4.2 [7–28]	13.1 ± 4.5 [7–23]	11.1 ± 4.1 [7–24]	9.3 ± 2.9 [7–27]
HADS‐A, 0–21, mean ± SD [range]	7.2 ± 4.4 [0–19]	10.0 ± 5.4 [0–21]	5.6 ± 3.7 [0–15]	3.9 ± 3.3 [0–18]
Depression diagnosis, *n* (%)	44 (46%)^b^	35 (36%)	21 (21%)	24 (12%)^c^
Dizziness characteristics				
VSS: total score, 0–60, mean ± SD [range]	13.2 ± 10.3^e^ [0–50]	21.7 ± 10.9^b^ [4–51]	7.0 ± 6.4^f^ [0–50]	2.9 ± 3.1^g^ [0–17]
VSS: vertigo subscale, 0–32, mean ± SD [range]	8.2 ± 7.3^e^ [0–31]	13.6 ± 7.3^b^ [1–31]	2.9 ± 3.5^f^ [0–28]	0.9 ± 1.8^f^ [0–15]
VSS: arousal subscale, 0–28, mean ± SD [range]	5.0 ± 4.1^e^ [0–20]	8.1 ± 5.2^b^ [0–23]	4.0 ± 3.6^f^ [0–22]	2.0 ± 2.1^f^ [0–8]

Abbreviations: ADL, activities of daily living; HADS‐A, anxiety subscale of the Hospital Anxiety Depression Scale; PPPD, persistent postural–perceptual dizziness; VSS, Vertigo Symptom Scale.

^a^ Missing data for two participants throughout, as they did not answer question on dizziness experience.

^b^ Two missing values.

^c^ One missing value.

^d^ Three missing values.

^e^ Five missing values.

^f^ Four missing values.

^g^ Ten missing values.

^h^ Twenty‐six missing values.

### Development of the Balance Vigilance Questionnaire

We adapted the existing Pain Vigilance and Awareness Questionnaire (PVAQ) [16] to balance/dizziness through patient and public involvement (PPI). PPI was conducted with 15 individuals living with a variety of balance disorders (including PPPD, benign paroxysmal positional vertigo [BPPV], undiagnosed chronic dizziness, and older adults with generalized imbalance) and four experienced clinicians with expertise in treating balance disorders. This reiterative process involved first adapting individual items from pain to balance/dizziness, and then removing any items that were now deemed irrelevant. Discussions revealed a number of important constructs relevant to balance vigilance that were not captured in the original PVAQ. Specifically, PPI members felt that some level of vigilance may be a normal response to balance problems, but that such vigilance may become maladaptive based on the subsequent emotional and behavioural consequences. It was therefore decided that additional items were needed to capture these seemingly important constructs (e.g., pervasive worry, fear avoidance). These items were developed through discussions with the PPI members. Wording of each item was refined through further discussion, and feedback on each iteration of the Balance Vigilance Questionnaire (Balance‐VQ) continued until consensus and agreement was reached.

This resulted in an 11‐item Balance‐VQ that was used for validation. This initial version of the scale can be viewed on the Open Science Framework repository for this project at https://osf.io/wq37x.

### Procedure

All participants completed an online survey hosted at the Jisc Online Surveys platform at baseline (T1). First, participants provided basic demographic information, self‐reporting whether they had a neurological, vestibular (including duration of dizziness symptoms), or psychiatric diagnosis, their education level, general health, number of medications, balance problems, number of falls in the past 12 months, and whether they require assistance for basic activities of daily living. As per previous related research [17], we also asked participants if they ever “experienced dizziness that was unrelated to alcohol or drug consumption or standing up too quickly.”

Participants then completed a battery of questionnaires. This included first the newly developed 11‐item Balance‐VQ. Participants were asked to score each item from 1 (never) to 5 (always), with respect to how they “typically feel in relation to their balance.” Next, they completed the Vertigo Symptom Scale–short form (VSS‐sf) [18]. This 15‐item scale assesses the frequency of vertigo, dizziness, and associated automatic arousal symptoms over the past month. Items are scored from 0 (never) to 4 (very often [most days]), with total scores thus ranging from 0 to 60. It has two subscales: one assessing vertigo symptoms (eight items) and another assessing autonomic arousal (seven items). Participants also completed the seven‐item anxiety subscale of the Hospital Anxiety and Depression Scale (HADS‐A), which assesses recent symptoms of anxiety [19]. Finally, they completed the seven‐item short version of the Falls Efficacy Scale–International (FES‐I) [20] to provide information on the degree of any concerns about falling experienced.

The first 125 participants were then invited to complete the Balance‐VQ again 2 weeks later (T2), for test–retest reliability. Here, they also reported whether they had experienced any falls, or serious worsening of balance or dizziness in the 2 weeks since first completing the Balance‐VQ. This served as our anchor; only participants that had not fallen and had not experienced serious worsening of balance/dizziness were included in the retest analyses.

### Statistical analysis

**FIGURE 1:**
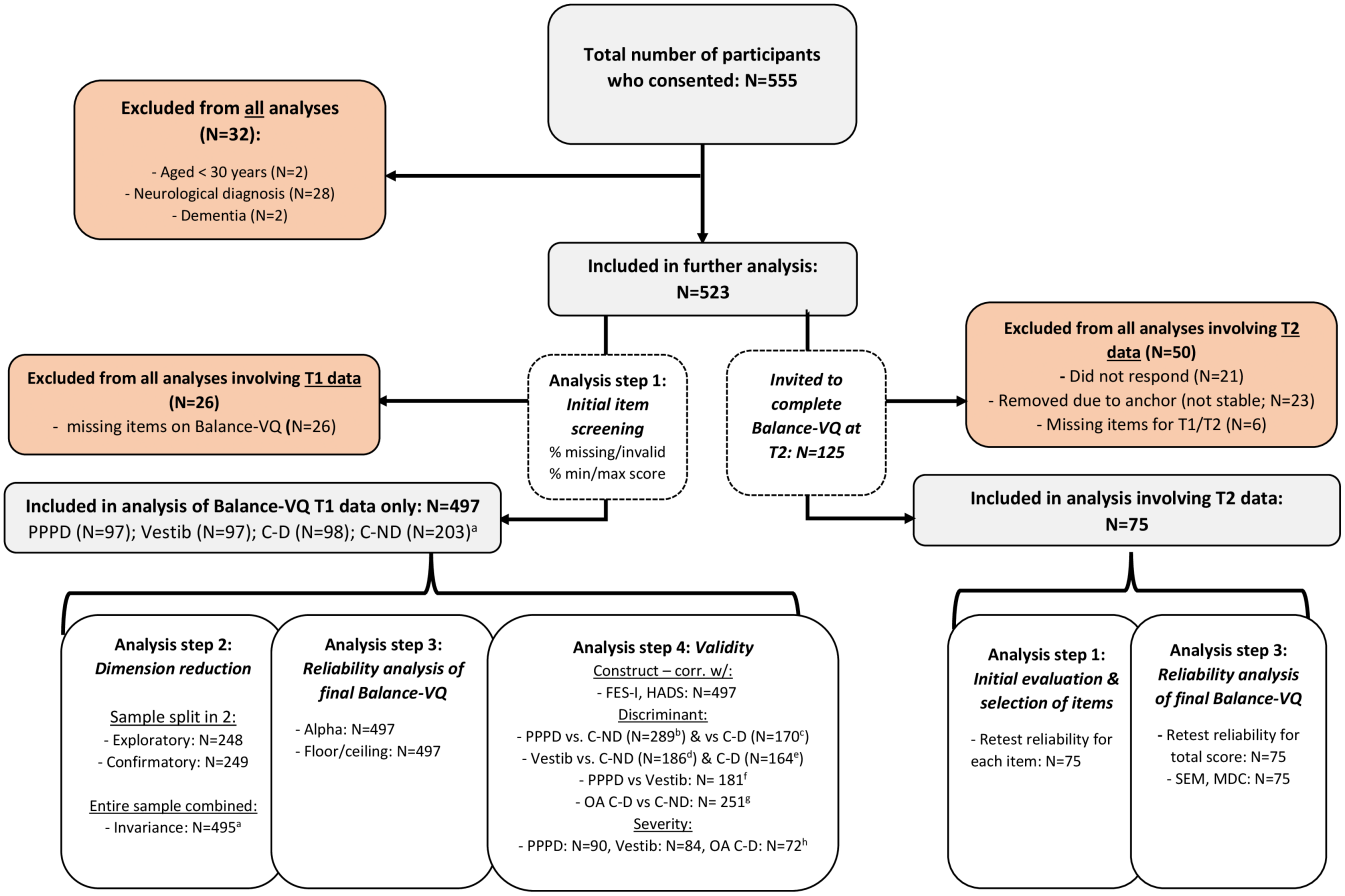
Study flow. ^a^Two participants failed to self‐report their dizziness experiences. These were removed only from analyses where subgrouping based on this variable was relevant. ^b–h^For these analyses, some participants were excluded due to missing data for at least one of the control variables: ^b^five PPPD and six C‐ND group members excluded; ^c^seven PPPD and 18 C‐D group members excluded; ^d^eight vestibular and six C‐ND members excluded; ^e^13 vestibular and 18 C‐D group members excluded; ^f^five PPPD and eight vestibular group members excluded; ^g^only the control group participants aged ≥60 years were included, of whom five older control–dizzy and seven older control–non‐dizzy participants were excluded; ^h^seven PPPD, 13 vestibular, and 15 older adult C‐D group members excluded. Balance‐VQ, Balance Vigilance Questionnaire; C‐D, controls without diagnosed neuro‐otological dysfunction, but with self‐reported dizziness; C‐ND, controls without diagnosed neuro‐otological dysfunction, and with no self‐reported dizziness; FES‐I, Falls Efficacy Scale–International; HADS, Hospital Anxiety and Depression Scale; MDC, minimal detectable change; OA, older adults; PPPD, persistent postural–perceptual dizziness; Vestib = vestibular diagnosis group.

All data were analysed with SPSS and AMOS (version 28; IBM, Chicago, IL). Unless stated otherwise, alpha was set at *p* = 0.05. Figure 1 summarizes the flow of the study and analyses, which broadly involved the following steps.

#### Step 1: Screening of individual items

Balance‐VQ items were considered for removal if (i) there were a large number of missing (or multiple) responses (>5%) at T1, (ii) >50% of responses at T1 were the minimum or maximum score, or (iii) an individual item's test–retest reliability was low (two‐way, random effect, consistency single measures intraclass correlation coefficient [ICC] < 0.5) [21].

#### Step 2: Dimension reduction and validation

Next, exploratory factor analysis and subsequent confirmatory factor analysis were performed. Participants were first randomly allocated (using random.org, 50:50 ratio) to either an "exploratory" or "confirmatory" subsample. We then first performed exploratory analysis using principal axis factoring (varimax rotation) using the T1 Balance‐VQ data for the "exploratory" subgroup. Visual inspection of the scree plot was done to identify the number of latent factors. Items were considered for removal if they loaded on multiple factors, loaded insufficiently (<0.4) [22] on a single factor, or exhibited low item‐rest (*r*'s < 0.3) and/or high interitem correlations (*r*'s > 0.7). This was followed by confirmatory factor analysis using the T1 Balance VQ data of the "confirmatory" subgroup (using maximum likelihood estimation) [23]. Items were constrained to load on the underlying factor(s) they had been associated with in the exploratory factor analysis. Pairs of error terms within a factor could covary if this improved model fit. We evaluated model fit using predefined criteria [24, 25, 26]; see Supplementary Material S3 for further details.

So‐called “measurement invariance” was determined to assess whether the scale structure was similar across participants from the PPPD, vestibular, and control groups with or without dizziness (i.e., four subgroups in total). For this analysis, we used T1 Balance‐VQ scores from the entire sample, and recommended criteria to evaluate changes in model fit for different levels of invariance [27]. See Supporting Information for details.

#### Step 3: Reliability and measurement error

In this step, we evaluated the finalized Balance‐VQ's internal consistency (Cronbach alpha) and test–retest reliability (two‐way, random effect, consistency, single measures ICC). For both alpha and ICC, we considered values > 0.70 to be satisfactory. Also, measurement error (SEM = SD + 2*√[1 − ICC]) [28] and minimal detectable change (MDC) were assessed on the group and individual level (MDC_group_ = SEM × 1.96 × √2/√n; MDC_individual_ = SEM × 1.96 × √2) [29]. Finally, floor and ceiling effects were determined (i.e., >15% of participants scoring lowest/highest possible summed scores [30]).

#### Step 4: Construct validity

Construct validity was assessed by correlating (Spearman rho) Balance‐VQ total scores with scores on (i) measures of balance‐specific (Falls‐Efficacy Scale) and (ii) generalized anxiety (HADS‐A). Construct validity would be evidenced if we would find significant weak to moderate correlations (0.3–0.5).

Furthermore, we used logistic regressions to assess whether Balance‐VQ scores were predictive of group status for groups in which balance vigilance has been implicated as a potential risk factor for dizziness, when controlling for important covariates (age, gender, generalized anxiety symptoms, depression diagnosis, number of medications). Specifically, we assessed whether people with higher Balance‐VQ scores would have greater odds of (i) PPPD group membership versus the control–no dizziness group membership, (ii) PPPD group membership versus vestibular group membership, (iii) PPPD group membership versus control–dizzy group membership, and (iv) experiencing dizziness versus no dizziness in daily life among control group members >60 years of age (as discussed in the Introduction, this is a group that frequently experiences "unexplained dizziness" proposed to also relate to balance vigilance [11, 12]). As a further control analysis, we also assessed the relationship between Balance‐VQ scores predicting vestibular group membership and both non‐dizzy and dizzy control group membership (because if the role of balance vigilance in maintaining dizziness is unique to PPPD, we would expect it not to distinguish between these other groups and for these analyses to be nonsignificant). These analyses were done in two steps, namely, only using Balance‐VQ scores as independent variable for the first step, followed by the addition of control variables in a second step. Note that when predicting group status between two "dizzy" groups (e.g., PPPD vs. vestibular group; PPPD vs. control–dizzy; vestibular vs. control–dizzy), we additionally controlled for severity of dizziness symptoms (VSS total score), in addition to the aforementioned covariates.

Finally, we conducted linear regressions to assess whether Balance‐VQ score was associated with dizziness severity (VSS total scores), when controlling for the same covariates as reported above. We did this analysis separately for the PPPD, vestibular, and control groups (for the latter focusing on older adults [>60 years] with dizziness only).

#### Step 5: Receiver operating characteristic analysis to identify cutoff values

Receiver operating characteristic (ROC) analyses were conducted to determine optimal cutoff points to determine (i) PPPD versus controls (non‐dizzy); (ii) PPPD versus controls (dizzy); (iii) older adult controls dizzy versus older adult controls non‐dizzy; and (iv) individuals meeting the cutoff point for anxiety using the HADS‐A (≥11/21) versus below this cutoff value, given the previously reported associations between anxiety and general bodily vigilance in balance disorders [9].

#### Sample size considerations

We aimed for an overall sample of 500 participants, to allow for two samples of 200 participants each for the exploratory and confirmatory factor analyses, respectively (exceeding the recommended subject‐to‐variable ratio of 10:1 [31]). For test–retest reliability analysis, power analysis showed that a sample size of 60 “stable” participants would be more than sufficient to be able to detect an ICC of 0.80 with a 95% confidence interval (CI) of 0.70–0.90.

#### Missing data

Participants with any missing data for the Balance‐VQ were excluded from all analyses (*n* = 26; see Supporting Information for information regarding missingness for individual Balance‐VQ items). Missing data rates for Short FES‐I, VSS‐sf, and HADS‐A were 0.2%, 0.8%, and 1.9%, respectively. As per recommendations [20, 32], missing items for the short FES‐I and the HADS‐A were handled using the individual‐mean imputation approach. (Note that this approach is recommended in cases where participants are missing data for either a single item [short FES‐I] or three items or fewer [HADS‐A]. These criteria were met for all missing data in the present research.) As there are no guidelines for handling missing data for the VSS‐sf, participants with any missing data were excluded from all analyses involving this scale (*n* = 33).

### Data availability and preregistration

Analyses and data‐handling procedures were preregistered (https://osf.io/d9hxn?view_only=true and https://osf.io/abxec). Data relevant to this project can be found here at https://osf.io/x4zph/. This project page also contains a document that details (justification for) any major deviations from the registered analysis protocol: https://osf.io/tgh6c.

## RESULTS

### Participant characteristics

Figure 1 summarizes the flow of the study. In total, 555 participants completed the study at T1. Of these, 32 were excluded because they did not meet inclusion criteria. Furthermore, as stated earlier, 26 participants had missing data for one or more items of the Balance‐VQ, and hence were excluded from all further analyses. Therefore, in total we included T1 data from 497 participants. Of these, 97 participants had a diagnosis of PPPD, and 97 had a current vestibular diagnosis other than PPPD. Breakdown of self‐reported diagnoses is as follows: labyrinthitis/vestibular neuritis, *n* = 29; Ménière disease, *n* = 19; vestibular migraine, *n* = 19; BPPV, *n* = 13; vestibular hypofunction, *n* = 2; mal de débarquement, *n* = 1; multiple, *n* = 8; unspecified vertigo, *n* = 6. Although the remaining 303 control participants were without diagnosed neuro‐otological dysfunction, 98 of these did self‐report dizziness complaints in daily life (control–dizzy), with 203 control participants reporting no dizziness (control–non‐dizzy). Participant characteristics are summarized for each of these four participant groups in Table 1.

Seventy‐five participants also completed the Balance‐VQ at T2. Of these, 13 had PPPD, 13 had a vestibular diagnosis that was not PPPD, 14 were classified as control–dizzy, and 35 were classified as control–non‐dizzy. Overall, the retest sample's characteristics were broadly similar to those of the overall (*n* = 497) sample (see Supporting Information S1 for a detailed overview).

### Initial screening and selection of items

We evaluated the performance of the individual items of the Balance‐VQ. There were no clear issues with missing items (*n* = 30 missing responses in total, *n* ≤ 7 (1.3%) for separate items). Evaluation of scoring distribution and of reliability indices revealed potential issues with item 9 (minimum value for 58% of participants) and with item 11 borderline floor (42%) and poor retest reliability (ICC = 0.549). These two items were therefore excluded from further analyses. Supporting Information S2 summarizes item‐level analysis results.

### Dimension reduction and validation

Exploratory factor analysis on the nine remaining items (items 1–8 and item 10), revealed a one‐factor solution (explained variance = 61.7%). With the exception of item 10, all items loaded on this factor (loadings ≥ 0.611; see Table 2). Item 10 was therefore removed from further analysis. Furthermore, we removed items 1 and 8 due to very high inter‐item correlations between item 1 and item 3 (*r* = 0.815), and between item 8 and items 1, 3, and 5 (*r*'s = 0.751–0.766). The analysis was run a second time without items 1, 8, and 10. Only one component was identified, explaining 66.9% of variance. All six items loaded highly on this component (0.618–0.850; see Table 2).

**TABLE 2:** Factor loadings for each item, presented separately for each of the two runs of the factor analysis.

Item	Run 1^a^	Run 2^b^ (after excluding items 1, 8, and 10)
	Factor loading (explained variance = 61.7%)	Factor loading (explained variance = 66.9%)
	Factor 1	Factor 1
1. I closely monitor how steady my balance feels	**0.827**	n/a
2. I become alarmed by sudden or temporary changes in steadiness	**0.722**	**0.850**
3. I am vigilant to small changes in how steady my balance feels	**0.866**	**0.830**
4. I immediately know when my balance worsens	**0.668**	**0.618**
5. When something happens that affects my balance, I am anxious to check how much my steadiness has decreased	**0.783**	**0.835**
6. I worry about fluctuations in steadiness	**0.751**	**0.846**
7. I avoid situations that I fear will affect my balance and make me less steady	**0.611**	**0.668**
8. I keep careful track of how steady my balance feels	**0.846**	n/a
10. I remain calm in situations that worsen my balance	0.071	n/a

*Note:* Bold values indicate loading of greater than 0.4 [22].

Abbreviations: KMO, Kaiser–Meyer–Olkin assessment; n/a, not applicable.

^a^ KMO = 0.917; all individual KMOs ≥.749 (>0.5 threshold; Field 2018 [31]).

^b^ KMO = 0.896; individual KMOs ≥ 0.882. With the exception of item 10, all items loaded on this factor. We removed item 10 from further analysis. Furthermore, we removed items 1 and 8 due to very high interitem correlations (0.751–0.815).

### Confirmatory factor analysis and measurement invariance

**FIGURE 2:**
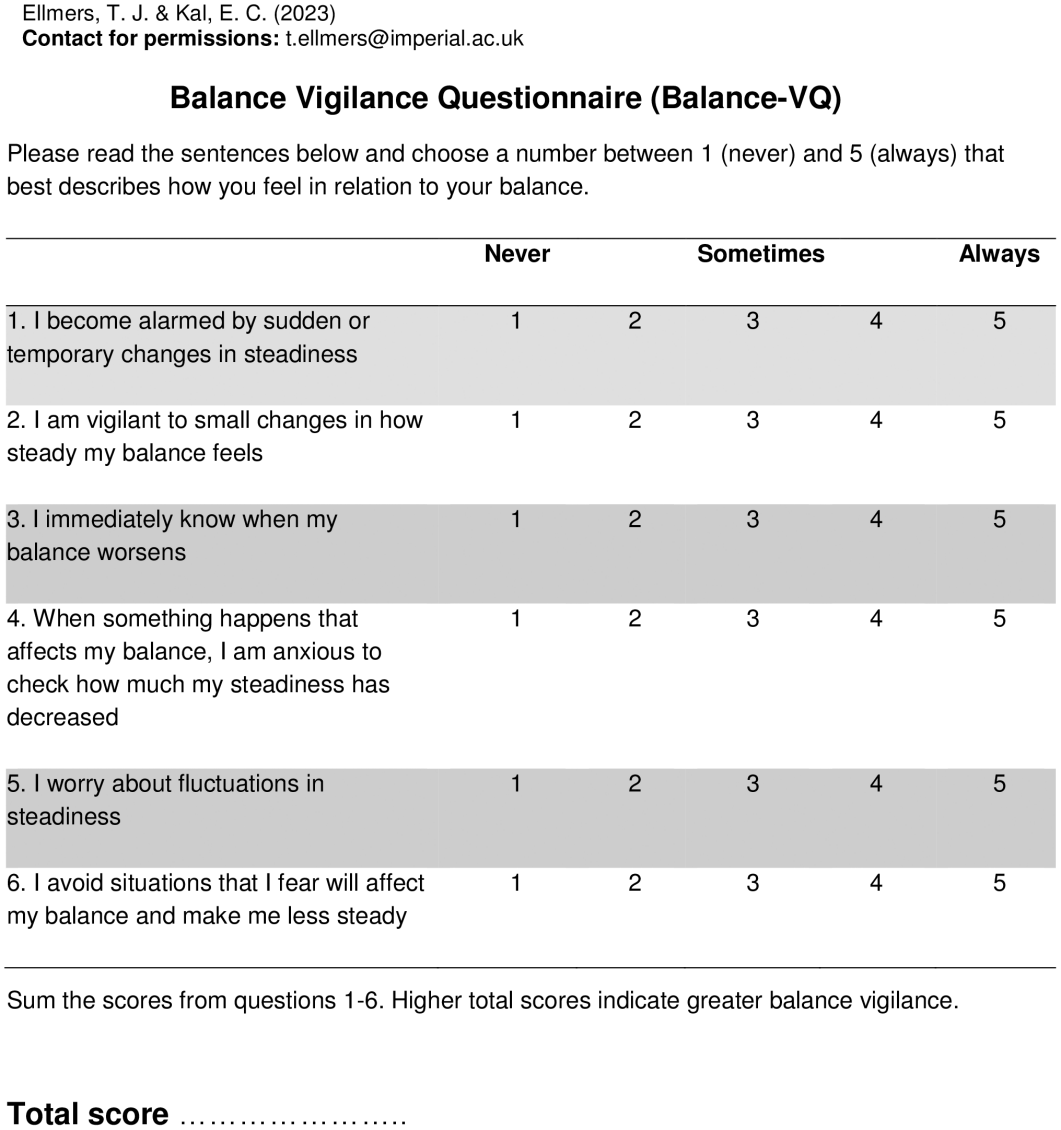
Final six‐item Balance Vigilance Questionnaire. Overall balance vigilance is scored by summing the scores for all items. Total scores range between 6 and 30, with higher scores indicating greater balance vigilance. A score of 19 or greater indicates high balance vigilance.

Overall, confirmatory factor analysis supported the one‐factor structure identified in the exploratory factor analysis. The model demonstrated sufficient measurement invariance (see Supporting Information S3), suggesting that the structure of the Balance‐VQ is similar for the different populations tested, namely, controls with or without dizziness experiences, people with a vestibular diagnosis, and people with a diagnosis of PPPD. Figure 2 presents the final Balance‐VQ.

### Reliability and measurement error analysis

The Balance‐VQ showed excellent internal consistency (α = 0.905) and test–retest reliability (ICC = 0.797, 95% CI = 0.697–0.867). The standard error of measurement was 2.93 points. MDC values were 0.9 (group level) and 8.1 (individual level). There were no clear floor or ceiling effects; 7.6% (*n* = 38) of individuals scored the minimal possible score (6 points), whereas 4.0% (*n* = 20) scored the maximal possible score (30 points).

### Construct validity analysis

#### Correlations of Balance‐VQ with other related constructs

Summed Balance‐VQ scores correlated with FES scores (*r* = 0.624, *p* < 0.001, 95% CI = 0.567–0.674, *n* = 497) and generalized anxiety (HADS‐A scores; *r* = 0.483, *p* < 0.001, 95% CI = 0.412–0.548, *n* = 497). Broadly speaking, this confirmed the hypothesis that the Balance‐VQ measures related but distinct constructs compared to these outcome measures, although the correlation with FES scores was somewhat higher than anticipated.

#### Associations with Balance‐VQ and participant group status

**FIGURE 3:**
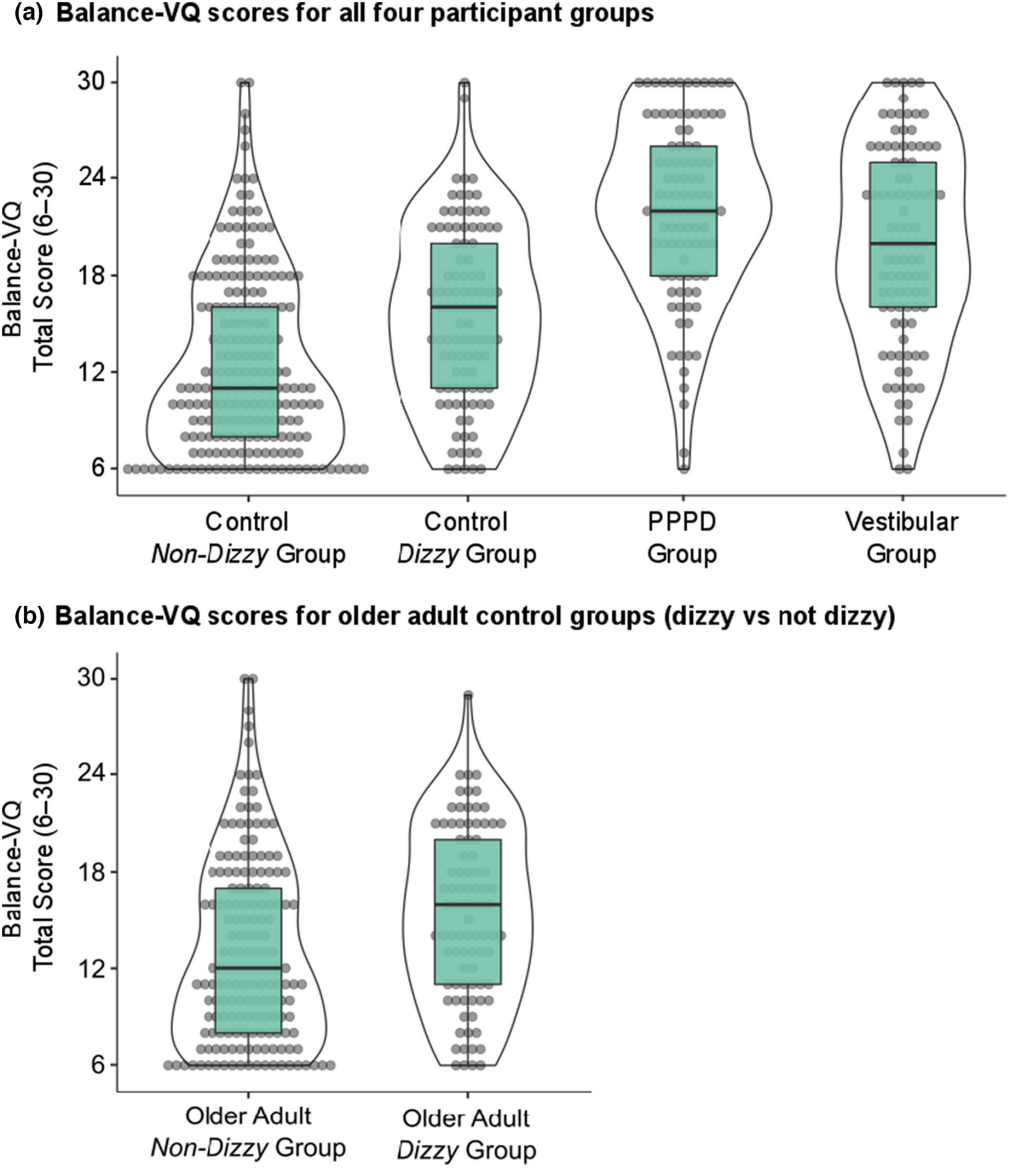
Total summed Balance Vigilance Scores (6–30) for all four participant groups (a), as well as presented separately for the older adults (≥60 years of age; b) in the non‐dizzy versus dizzy control groups. Note that the "Vestibular" group consists of individuals diagnosed with a vestibular disorder other than persistent postural–perceptual dizziness (PPPD), whereas the "Control Dizzy" group consists of individuals without diagnosed neuro‐otological dysfunction who nonetheless experience some degree of dizziness. Median, interquartile range, and individual scores are presented.

Mean scores for the control–non‐dizzy, control–dizzy, PPPD, and vestibular groups at T1 are presented in Figure 3a. As we also made specific comparisons with the controls ≥60 years of age, their data can be observed in Figure 3b.

In short, higher Balance‐VQ scores were associated with greater odds of being in the PPPD group versus both the control–non‐dizzy group (odds ratio [OR] = 1.34, 95% CI = 1.23–1.46) and the control–dizzy group (OR = 1.15, 95% CI = 1.00–1.32), even when controlling for potential confounding factors. Similarly, higher Balance‐VQ scores were independently associated with greater odds of being in the vestibular group versus both the control–non‐dizzy group (OR = 1.22, 95% CI = 1.16–1.28) and the control–dizzy group (OR = 1.13, 95% CI = 1.07–1.20). Also, for older adult control participants, higher Balance‐VQ scores were independently associated with greater odds of reporting dizziness in daily life (OR = 1.06, 95% CI = 1.01–1.12). Although Balance‐VQ scores were associated with greater odds of having PPPD rather than a vestibular diagnosis in the univariable analysis (OR = 1.08, 95% CI = 1.02–1.13), these scores no longer discriminated between the two groups when controlling for confounding factors, including dizziness severity (OR = 0.99, 95% CI = 0.92–1.07).

Please see Table 3 for full results of analyses involving the PPPD group. For detailed results of the logistic regression analyses comparing the vestibular group to control–dizzy and control–non‐dizzy, and comparing older adult control participants, please see Supporting Information S4.

**TABLE 3:** Full results of analyses involving the PPPD group.

	Model 1: PPPD versus control–non‐dizzy^a^		Model 2: PPPD versus control–dizzy^b^		Model 3: PPPD versus vestibular^c^	
	*p*	Odds ratio [95% CI]	*p*	Odds ratio [95% CI]	*p*	Odds ratio [95% CI]
Step 1						
Intercept						
Balance‐VQ	**<0.001**	1.32 [1.24–1.41]	**<0.001**	1.23 [1.15–1.32]	**0.007**	1.08 [1.02–1.13]
Step 2						
Intercept						
Balance‐VQ	**<0.001**	1.34 [1.23–1.46]	**0.042**	1.15 [1.00–1.32]	0.857	0.99 [0.92–1.07]
Age in years	**<0.001**	0.90 [0.87–0.94]	**<0.001**	0.86 [0.81–0.91]	**<0.001**	0.93 [0.90–0.95]
Gender, reference = male	**0.045**	3.33 [1.03–10.80]	0.529	1.67 [0.34–8.24]	0.122	0.40 [0.13–1.28]
HADS‐A	**0.022**	1.13 [1.02–1.26]	0.348	1.06 [0.93–1.21]	0.173	1.06 [0.98–1.14]
Depression diagnosis, reference = no	**<0.001**	7.24 [2.26–23.18]	0.400	1.80 [0.46–7.06]	0.132	0.56 [0.26–1.19]
No. of medications > 4, reference = no	0.217	0.37 [0.08–1.79]	0.982	0.98 [0.15–6.33]	0.304	0.55 [0.18–1.71]
VSS total	NA^d^	NA	**0.003**	1.17 [1.05–1.29]	**0.016**	1.05 [1.01–1.10]

*Note*: **Model 1**, Step 1: Nagelkerke *R* ^2^ = 0.53, χ^2^(1) = 138.74, *p* < 0.001, AUC = 0.89, specificity = 0.85, sensitivity = 0.75, cutoff = 0.375. Step 2: Nagelkerke *R* ^2^ = 0.79, χ^2^(6) = 240.49, *p* < 0.001, AUC = 0.97, specificity = 0.90, sensitivity = 0.89, cutoff = 0.30. **Model 2**, Step 1: Nagelkerke *R* ^2^ = 0.37, χ^2^(1) = 54.85, *p* < 0.001, AUC = 0.81, specificity = 0.75, sensitivity = 0.67, cutoff = 0.59. Step 2: Nagelkerke *R* ^2^ = 0.83, χ^2^(7) = 165.97, *p* < 0.001, AUC = 0.98, specificity = 0.94, sensitivity = 0.93, cutoff = 0.49. **Model 3**, Step 1: Nagelkerke *R* ^2^ = 0.06, χ^2^(1) = 7.83, *p* = 0.005, AUC = 0.62, specificity = 0.60, sensitivity = 0.50, cutoff = 0.54. Step 2: Nagelkerke *R* ^2^ = 0.44, χ^2^(7) = 69.71, *p* < 0.001, AUC = 0.84, specificity = 0.76, sensitivity = 0.76, cutoff = 0.53. Bold values indicate statistical significance (*p* < 0.05).

Abbreviations: AUC, area under the curve; Balance‐VQ, Balance Vigilance Questionnaire; CI, confidence interval; HADS‐A, anxiety subscale of Hospital Anxiety and Depression Scale; NA, not applicable; PPPD, persistent postural–perceptual dizziness; VSS = Vertigo Symptom Scale (total score).

^a^ Five PPPD and six control group members could not be included due to missing values for one or more of the control variables.

^b^ Eighteen control–dizzy and seven PPPD group members could not be included due to missing values for one or more of the control variables.

^c^ Seven PPPD and 13 vestibular group members could not be included due to missing values for one or more of the control variables.

^d^ As VSS scores (i.e., dizziness severity) are a proxy for group membership in PPPD versus non‐dizzy controls, this variable was not entered in Model 1 (but only Model 2 and 3, where we were comparing two separate "dizzy" groups).

#### Associations with Balance‐VQ scores and dizziness severity

Finally, in PPPD, vestibular, and older adult control–dizzy groups, Balance‐VQ scores were significantly associated with VSS scores, even when controlling for confounding variables (PPPD: β = 0.83, 95% CI = 0.43–1.22, *p* < 0.001; vestibular: β = 0.54, 95% CI = 0.20–0.88, *p* = 0.002; control–dizzy: β = 0.33, 95% CI = 0.14–0.52, *p* < 0.001). This association appeared the strongest for the PPPD group, not only with respect to the β value (adjusted for confounding variables) reported above, but also because adding the confounding variables did not significantly increase the model fit. See Supporting Information S5 for detailed results for each group.

#### Cutoff values to identify high balance vigilance

Area under the curve scores were (i) 0.88 for PPPD versus controls (non‐dizzy), (ii) 0.80 for PPPD versus controls (dizzy), (iii) 0.63 for older adult controls (dizzy) versus older adult controls (non‐dizzy), and (iv) 0.78 for individuals meeting the cutoff point for anxiety using the HADS‐A versus below this cutoff value. Based on these analyses, we defined the cutoff point of ≥19/30 on the Balance‐VQ to identify someone as having high balance vigilance (as this value represented the best balance between sensitivity and specificity, across these four analyses). Please see Supporting Information S6 for ROC curve plots and corresponding coordinate tables.

## DISCUSSION

The aim of this research was to develop (through patient input) a validated balance‐specific measure of vigilance that comprehensively assesses the varied ways in which this construct may manifest. Our findings confirm that the Balance‐VQ is a valid and reliable self‐report instrument for assessing vigilance towards balance. A recent review exploring factors predicting the development of PPPD and chronic dizziness recommended that clinicians screen for bodily vigilance to identify the patients most at risk of developing symptoms of chronic dizziness following an acute vestibular insult [8]. We propose that the short, six‐item Balance‐VQ (with the established cutoff point of ≥19/30) would be well suited for such purpose.

As hypothesized, Balance‐VQ scores were higher in individuals diagnosed with PPPD compared to controls (without dizziness), and scores predicted group membership even when controlling for confounding variables such as age, gender, and anxiety. These findings support previous work highlighting the role of generalized bodily vigilance in the development and maintenance of PPPD [5, 6, 7, 8, 9]. Additionally, Balance‐VQ scores differentiated between individuals diagnosed with PPPD and healthy "controls" who nonetheless experience dizziness in daily life (despite having no diagnosed neuro‐otological dysfunction). This result remained even when controlling for dizziness severity (and other additional confounds). This may suggest that balance vigilance is not merely a consequence of experiencing symptoms of dizziness, but rather relates specifically to symptoms of PPPD.

On the other hand, although Balance‐VQ scores were higher in the PPPD group compared to individuals with diagnosed vestibular disorders other than PPPD, Balance‐VQ scores did not discriminate between the two groups when confounding factors (including dizziness severity) were controlled for. However, a major limitation of the present work was the remote nature of the data collection (due to COVID‐19 restrictions on face‐to‐face testing). This meant that we were not able to conduct objective neuro‐otological testing, and also had to rely on self‐reported diagnosis. Consequently, we propose that the capability of the Balance‐VQ to distinguish between PPPD and controls with/without dizziness—but not between PPPD and other vestibular patients—could perhaps be due to the presence of undiagnosed symptoms of PPPD in the non‐PPPD vestibular group. A number of these individuals experienced continued symptoms of dizziness, despite initially being diagnosed with an acute peripheral disorder (BPPV or vestibular neuritis) many months or even years previously (indicating the development of a secondary vestibular disorder, such as PPPD). Further, approximately 20% of the 'vestibular' sample had vestibular migraine, a disorder known to frequently coexist with PPPD symptoms [33]. Future work should therefore look to compare Balance‐VQ scores between individuals diagnosed with PPPD and a group of individuals diagnosed with a uniform and well‐defined peripheral vestibular disorder (e.g., bilateral vestibular hypofunction).

Although the present study was cross‐sectional rather than prospective in nature, prospective designs have identified generic (i.e., not specific to balance) vigilant monitoring of bodily signals as a strong risk factor for the development of PPPD [9]. Given the generic nature of the tools used to previously explore this relationship, we propose that the more specific assessment of balance vigilance developed in the present work may be an even more sensitive measure for predicting the development of chronic dizziness. This could then help identify which patients may be most likely to benefit from rehabilitation that specifically targets attention during balance, a strategy that seems particularly effective for improving symptoms in PPPD (see Herdman et al. [34]). Future work should look to explicitly test the sensitivity of the Balance‐VQ for predicting the development of chronic dizziness (particularly following an acute neuro‐otological insult).

Although the specific mechanism through which balance vigilance may contribute to symptoms of chronic dizziness remains unknown, there is a wealth of evidence that describes how consciously attending sensory input can alter the way in which the brain processes these signals (e.g., Veldhuijzen et al. [35], Seminowicz et al. [36], Little and Woollacott [37]). Some researchers have hypothesized that heightened monitoring of balance in PPPD may therefore amplify the neural processing of discrepancies between anticipated and actual postural feedback signals (i.e., "prediction errors") [6, 7]. This could then lead to individuals becoming aware of minor changes in postural sway that are always occurring (see Carpenter et al. [38]), but that typically take place outside of conscious awareness given that the "error signals" are so low [39]. As Van den Bergh et al. [40] write, “if there are no cues directing attention to the body, minor prediction errors may go unnoticed” (p. 195). Supporting this stance, recent research highlights that conditions that increase the amount of attention directed towards consciously monitoring balance lead to the cortical processing of minor changes in postural stability that would otherwise be largely ignored at the cortical level [41].

Similar mechanisms have also been proposed to contribute to the distorted perceptions of instability that are common in older adults [10, 42], particularly in those with "unexplained dizziness" [11, 43]. In line with these suggestions, Balance‐VQ scores were higher in older adults without diagnosed neuro‐otological dysfunction but who nonetheless experienced dizziness and perceived themselves to be unstable. A general sensitivity for changes in bodily signals related to balance may serve an adaptive purpose in individuals with imbalance. But persistent active scanning to detect cues for one's physical condition may change the way that the brain processes this information, serving to exacerbate symptom perception, particularly when this leads to pervasive worries when symptoms are detected [44]. However, future research should look to scrutinize the specific mechanisms through which balance vigilance contributes to perceptions of instability and chronic dizziness across different populations.

Balance‐VQ scores correlated to dizziness severity (as measured by the VSS‐sf) in all three "dizzy" groups: PPPD, diagnosed vestibular disorder other than PPPD, and the "dizzy–control" group (those without diagnosed neuro‐otological dysfunction who nonetheless experience some degree of dizziness in daily life). However, based on CIs and mean estimates, this association was strongest for the PPPD group. This could be meaningful, as it indicates that the relationship between dizziness and balance vigilance may differ across types of dizziness. Alternatively, it could be an artefact of the PPPD group having, on average, more severe symptoms of dizziness. Future work should therefore look to explore this association further. Interestingly, Balance‐VQ scores were also high in a number of control individuals without dizziness, with approximately 15% scoring above the identified cutoff point for high balance vigilance (≥19/30). Like other symptoms and factors associated with PPPD (e.g., visual motion sensitivity) [17], this suggests that balance vigilance might exist on a spectrum in the general population. Whether high scores on the Balance‐VQ then predispose an individual to developing chronic dizziness following an acute neuro‐otological insult should be explored in future work.

A further limitation of this work relates to our recruitment strategy. We recruited four quite distinct groups of participants, creating a relatively heterogeneous overall sample used for the factor analyses. However, our results showed that the scale demonstrates “measurement invariance,” meaning that the structure of the Balance‐VQ holds similar across all groups (and that scores can be validly compared between these).

In summary, the short, six‐item Balance‐VQ presented herein was shown to be a valid and highly reliable tool to assess vigilance towards balance. Given the links between balance vigilance and chronic dizziness [6, 8], the Balance‐VQ could be a useful tool to help identify patients most likely to benefit from rehabilitation that specifically targets attention during balance (see Herdman et al. [34]). Future work should investigate the prospective utility of the scale for identifying those most at risk of developing chronic dizziness (e.g., PPPD).

## AUTHOR CONTRIBUTIONS

**Ellmers Toby J.:** Conceptualization; investigation; funding acquisition; writing – original draft; methodology; data curation. **Elmar C. Kal:** Conceptualization; methodology; validation; visualization; writing – review and editing; formal analysis.

## CONFLICT OF INTEREST STATEMENT

Neither of the authors has any conflict of interest to disclose.

## Supporting information


DATA S1

## Data Availability

The data that support the findings of this study are openly available in Open Science Framework at https://osf.io/x4zph/, reference number: x4zph.
