# Supplementary material for: Exploring the role of attention towards balance in chronic dizziness: Development of the Balance Vigilance Questionnaire
Source: Eur J Neurol. 2023 Nov 28;31(3):e16148. doi: 10.1111/ene.16148 (PMC11235928; doi:10.1111/ene.16148)
Supplement: Supplementary file 1 — DATA S1 [file ENE-31-e16148-s001.docx]

**Supplementary Material 1. Characteristics of Test-Retest Sample (total N=75)**

|  | **Vestibular  *(N = 13)*** | **PPPD *(N = 13)*** | **Controls  *(no neuro-otological dysfunction; N = 49)*** | |
| --- | --- | --- | --- | --- |
|  |  |  | *Control-dizzy (N=14)* | *Control-not dizzy (N=35)* |
| **Background / General Health** |  |  |  |  |
| Age in years (mean ± SD [range]) | 64.1 ± 11.2  [44-80] | 45.4 ± 11.3  [31-60]^b^ | 70.5 ± 8.6  [59-85]^a^ | 67.3 ± 15.4  [30-87] |
| Female gender (n; %) | 12 (92%) | 10 (77%) | 8 (57%) | 17 (49%) |
| Education Level – college / sixth form or above (n, %) | 12 (92%) | 10 (77%) | 11 (84%) | 30 (86%) |
| General health – self-reporting very good to excellent health (n; %) | 2 (15%) | 1 (8%) | 10 (71%) | 21 (60%) |
| Medication use >4 (n; %) | 2 (15%) | 2 (15%) | 1 (7%) | 5 (15%) |
| Peripheral neuropathy (n; %) | 0 (0%) | 0 (0%) | 0 (0%) | 1 (3%) |
| Diabetes (n; %) | 0 (0%) | 1 (8%) | 1 (7%) | 4 (11%) |
| **Physical functioning** |  |  |  |  |
| Falls in past 12 months (n; %) | 6 (46%) | 3 (23%) | 5 (36%) | 10 (29%) |
| Balance problems (n; %) | 11 (85%) | 10 (77%) | 6 (43%) | 5 (14%) |
| Walking aid (n; %) | 1 (8%) | 1 (8%) | 2 (14%) | 1 (3%) |
| ADL-assistance (n; %) | 1 (8%) | 1 (8%) | 0 (0%) | 0 (0%)^a^ |
| **Psychological functioning** |  |  |  |  |
| Short Falls Efficacy Scale – International  (7-28; mean ± SD [range]) | 13.2 ± 3.5  [8-20] | 13.0 ± 4.4  [8-21] | 9.6 ± 2.5  [7-15] | 9.4 ± 4.0  [7-27] |
| HADS-Anx  (0-21; mean ± SD [range]) | 7.1 ± 3.8  [1-15] | 10.1 ± 5.5  [0-20] | 4.9 ± 3.1  [0-12] | 4.6 ± 3.8  [0-18] |
| Depression Diagnosis (n; %) | 4 (31%) | 7 (54%) | 3 (21%) | 3 (9%) |
| **Dizziness characteristics** |  |  |  |  |
| VSS – Total score  (0-56; mean ± SD [range]) | 11.3 ± 8.0^a^  [2-25] | 18.3 ± 10.1 [6-44] | 7.3 ± 4.6^a^  [1-18] | 2.0 ± 2.2^b^  [0-8] |
| VSS – Vertigo Subscale (0-28) *(Mean ± SD [range])* | 6.8 ± 5.3^a^  [1-18] | 11.5 ± 8.8 [1-30] | 2.9 ± 2.1^a^  [0-7] | 0.5 ± 0.8^b^  [0-2] |
| VSS – Arousal Subscale (0-28) *(Mean ± SD [range])* | 4.5 ± 3.5^a^  [0-12] | 6.9 ± 4.2 [2-16] | 4.4 ± 3.5^a^  [1-12] | 1.6 ± 1.8^b^  [0-7] |

**NB:** ^a^1 missing value; ^b^2 missing values;

**Abbreviations:** ADL: Activities of Daily Living; HADS-Anx: Anxiety subscale of the Hospital Anxiety Depression Scale; SD: Standard Deviation; VSS: Vertigo Symptom Scale;

| **Table S2.** Initial screening of items. | | | | |
| --- | --- | --- | --- | --- |
| ***Item*** | ***% missing / n*** | ***% min/max score*** | ***ICC (95% CI)*** | ***Included?*** |
| 1. I closely monitor how steady my balance feels | 0.4% / 2 | 21% / 15% | .633  (.476, .752) | Yes |
| 2. I become alarmed by sudden or temporary changes in steadiness | 0.2% / 1 | 28% / 12% | .642  (.486, .758) | Yes |
| 3. I am vigilant to small changes in how steady my balance feels | 0.2% / 1 | 25% / 17% | .669 (.522, .777) | Yes |
| 4. I immediately know when my balance worsens | 0.6% / 3 | 17% / 32% | .595  (.427, .724) | Yes |
| 5. When something happens that affects my balance, I am anxious to check how much my steadiness has decreased | 0.6% / 3 | 34% / 13% | .700  (.563, .799) | Yes |
| 6. I worry about fluctuations in steadiness | 1.0% / 5 | 35% / 14% | .673  (.527, .780) | Yes |
| 7. I avoid situations that I fear will affect my balance and make me less steady | 0.6% / 3 | 27% / 12% | .725  (.597, .817) | Yes |
| 8. I keep careful track of how steady my balance feels | 0.8% / 4 | 36% / 10% | .576  (.403, .709) | Yes |
| 9. I become preoccupied with monitoring my level of steadiness | 0.2% / 1 | 58% / 5% | .609  (.444, .734) | No |
| 10. I remain calm in situations which worsen my balance | 1.3% / 7 | 26% / 7% | .690  (.550, .792) | Yes |
| 11. I dwell on my balance | 0.0% / 0 | 46% / 6% | .549  (.370, .690) | No |

**Supplementary Material 2. Results of initial screening of items.**

Results for step 1 of the analysis: item-level screening based on missing values, floor/ceiling effects, and retest reliability. Items 9 and 11 were excluded from further analysis based on this analysis. Note that item 11 technically met all required thresholds, but the combination of relatively low ICC value and a high proportion of people scoring minimum score made us decide to remove this item.

**NB:** Predetermined cut-off values were 5% (missing cases per item), 50% (% of maximal / minimal scores for an item), and ICC<.50. Excluded items - items 9 and 11 - are highlighted in red.

**Supplementary Material 3. Factor Analyses**Confirmatory factor analysis was performed using T1 data from the ‘confirmatory analysis subsample’ to evaluate model fit of a model where items 2-7 were constrained to load on one underlying factor/construct – i.e., the outcome from the exploratory analysis’ results. We evaluated model fit through assessment of: Standardised item-factor loadings, the chi-square statistic – both raw (χ^2^) and divided by its degrees of freedom (χ^2^/df; both should be close to zero for good fit), goodness-of-fit and comparative fit indices (CFI; values>.95 and values>.90 indicate good and acceptable fit), standardized root mean squared residual (SRMR; values<.08 indicate good fit), and the root mean square error of approximation (RMSEA; values<.05 and values<.08 indicate good and acceptable fit, respectively).^[[1]](#footnote-1)^

In an initial run, standardised item-factor loadings were all positive and high (.63-.91), with model fit indices showing mixed results (χ^2^(9)=52.869, p<.001; χ^2^/df=5.874; CFI=.957; GFI=.931; RMSEA=.140 [.105, .178]; SRMR=0.041). Inspection of the modification indices revealed that model fit could be substantially improved by allowing the residual error terms of items 3 and 4 to covary (MI=28.502). This was done in a second run. Item-factor loadings were again high and positive for each item (.60-.91), and model fit was largely satisfactory (χ^2^(8)=22.371, p=.004; χ^2^/df=2.796; CFI=.986; GFI=.970; RMSEA=.085 [.044, .128]; SRMR=0.028). Further inspection of the modification indices led us to the decision to also allow covariance between the residual error terms of items 4 and 5 (MI=6.620). This resulted in the final model as presented in Figure S3. Standardised item-factor loadings remained high (.59-.92), while model fit indices were now all acceptable to good (χ^2^(7)=15.558, p=.029 ; χ^2^/df=2.223; CFI=.992; GFI=.979; RMSEA=.070 [.021, .117]; SRMR=0.023).


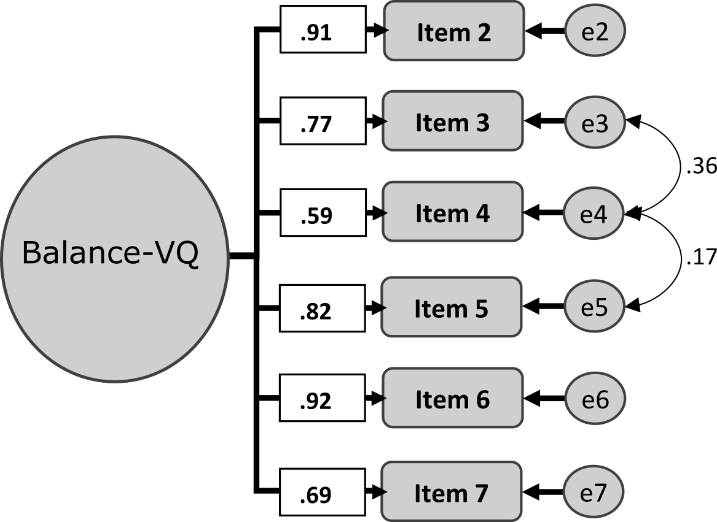


**Figure S3.** Final overall model yielded by the confirmatory factor analysis. Shown are the standardized item-factor loadings Abbreviated item numbers refer to the 6 selected items of the Balance-VQ). Also shown are the covariance between the residual error terms (abbreviated as ‘e’) of items 3 and 4, and 4 and 5.

**Measurement invariance testing**Table S3 shows the results of measurement invariance testing. To assess this, we evaluated model fit when item-factor loadings were free to differ between the 4 different participant subgroups (configural invariance), when item-factor loadings were equated across these groups (metric invariance), and when both the item-factor loadings and the intercepts of the model were equated across groups (scalar invariance). As shown in Table S3, we found evidence of sufficient configural, metric and scalar measurement invariance. Thus, the structure of the Balance-VQ seems similar across the different populations tested: i.e., controls with or without dizziness experiences, people with a vestibular diagnosis, and people with a diagnosis of PPPD.

| **Invariance test** | **χ^2^** | **χ^2^/df** | **CFI** | **RMSEA (90%CI)** | **SRMR** | **Model comp.** | **∆χ^2^** | **∆CFI** | **∆RMSEA**  **∆SRMR** | **Decision** |
| --- | --- | --- | --- | --- | --- | --- | --- | --- | --- | --- |
| **1. Config.** | 106.341  df=41  *p*<.001 | 2.594 | .944 | .064  [.049, .079] | .075 | N/A | N/A | N/A | N/A | **Accept** |
| **2. Metric** | 125.803 df=51  *p*<.001 | 2.467 | .936 | .061  [.048, .075] | .069 | 1 | 19.462 df=10  *p*=.067 | -.008 | -.003  -.007 | **Accept** |
| **3. Scalar** | 128.375 df=53  *p*<.001 | 2.399 | .935 | .060 [.047, .080] | .076 | 2 | 2.572 df=2  *p*=0.429 | -.001 | -.001 .007 | **Accept** |

**Table S3.** Results of measurement invariance testing.

**Abbreviations**: CFI = Comparative fit index; Config. = Configural; GFI = Goodness-of-fit index; Model comp. = Model comparison; N/A= Not applicable; RMSEA = Root mean square error of approximation; SRMR = Standardized root mean squared residual; df = degrees of freedom; **NB1**: Values presented are the mean estimates based on 100 subsampling analyses, for which we had randomly selected control group participants who were without dizziness for each subsample (mean N = 101, range: 84-121), to ensure that sample sizes were similar across the 4 groups; **NB2:** Configural invariance was deemed acceptable if CFI>.90, and if RMSEA and SRMR<.08. For metric and scalar invariance, acceptable model fit change indices were considered to be ∆CFI<-0.010, ∆RMSEA<0.015, and ∆SRMR<0.030 (metric invariance) or ∆SRMR<0.010 (scalar invariance);^[[2]](#footnote-2),^^[[3]](#footnote-3)^

**Supplemental Material 4.** Results of the logistic and linear regression analysis.

**Table S4A.** Results of logistic regression to determine association between Balance-VQ and dizziness status at T1 (Vestibular diagnosis = 1, N=89; vs Control not-dizzy = 0, N=197).^a^

|  | ***p*** | **Odds Ratio [95% CI]** |
| --- | --- | --- |
| **Step 1** | | |
| *Intercept* |  |  |
| Balance-VQ | **<.001** | 1.22 [1.16, 1.28] |
| **Step 2** | | |
| *Intercept* |  |  |
| Balance-VQ | **<.001** | 1.19 [1.12, 1.26] |
| Age *(in years)* | **.040** | 0.98 [0.95, 1.00] |
| Gender *(reference = male)* | **.005** | 3.75 [1.49, 9.42] |
| HADS – Anxiety | **.046** | 1,10 [1.00, 1.22] |
| Depression diagnosis *(reference = no)* | **<.001** | 5.26 [2.46, 11.25] |
| No. of medications > 4 *(reference = no)* | .438 | 0.68 [0.26, 1.79] |
| VSS Total | NA^b^ | NA |

**NB:** ^a^ 6 control not-dizzy and 8 Vestibular group members could not be included due to missing values for one or more of the control variables; ^b^ As VSS scores (i.e. dizziness severity) is a proxy for group membership between vestibular vs. non-dizzy controls, this variable was not entered; **NB2:** Step 1: Nagelkerke R^2^=.35; χ^2^(1)=82.56, p<.001; AUC = .70, Specificity = .76, Sensitivity = .71, cut-off: .33; Step 2: Nagelkerke R^2^=.51; χ^2^(6)=128.35, p<.001; AUC = .88, Specificity = .80, Sensitivity = .79, cut-off: .28; **Abbreviations:** Balance-VQ = Balance Vigilance Questionnaire; CI = Confidence interval; HADS = Hospital Anxiety and Depression Scale; VSS = Vertigo Symptom Scale (total score).

**Table S4B.** Results of logistic regression to determine association between Balance-VQ and dizziness status at T1 (Vestibular diagnosis = 1, N=84; vs Control-dizzy = 0, N=80).^a^

|  | ***p*** | **Odds Ratio [95% CI]** |
| --- | --- | --- |
| **Step 1** | | |
| *Intercept* |  |  |
| Balance-VQ | **<.001** | 1.13 [1.07, 1.20] |
| **Step 2** | | |
| *Intercept* |  |  |
| Balance-VQ | **.010** | 1.10 [1.02, 1.18] |
| Age *(in years)* | **.023** | 0.96 [0.93, 0.99] |
| Gender *(reference = male)* | .228 | 1.92 [0.67, 5.52] |
| HADS – Anxiety | .606 | 0.97 [0.88, 1.08] |
| Depression diagnosis *(reference = no)* | .116 | 1.87 [0.86, 4.09] |
| No. of medications > 4 *(reference = no)* | .354 | 0.64 [0.24, 1.66] |
| VSS – Total | .087 | 1.06 [0.99, 1.12] |

**NB:** ^a^ 18 control-dizzy and 13 Vestibular group members could not be included due to missing values for one or more of the control variables; **NB2:** Step 1: Nagelkerke R^2^=.17; χ^2^(1)=21.96, p<.001; AUC = .70, Specificity = .65, Sensitivity = .64, cut-off: .525; Step 2: Nagelkerke R^2^=.32; χ^2^(7)=44.41, p<.001; AUC = .78, Specificity = .72, Sensitivity = .70, cut-off: .485; **Abbreviations:** Balance-VQ = Balance Vigilance Questionnaire; CI = Confidence interval; HADS = Hospital Anxiety and Depression Scale; VSS = Vertigo Symptom Scale (total score);

**Table S4C.** Results of logistic regression to determine association between Balance-VQ and dizziness status at T1 (Control-dizzy (≥60 years) = 1, N=82; vs Control-not dizzy (≥60 years) = 0, N=160).^a^

|  | ***p*** | **Odds Ratio [95% CI]** |
| --- | --- | --- |
| **Step 1** | | |
| *Intercept* |  |  |
| Balance-VQ | **.002** | 1.08 [1.03, 1.13] |
| **Step 2** | | |
| *Intercept* |  |  |
| Balance-VQ | **.027** | 1.06 [1.01, 1.12] |
| Age *(in years)* | .221 | 0.97 [0.93, 1.02] |
| Gender *(reference = male)* | .270 | 1.48 [0.74, 2.99] |
| HADS – Anxiety | **.009** | 1.14 [1.03, 1.25] |
| Depression diagnosis *(reference = no)* | .601 | 1.23 [0.57, 2.65] |
| No. of medications > 4 *(reference = no)* | .835 | 0.92 [0.42, 2.00] |
| VSS Total | NA^b^ | NA |

**NB:** ^a^ 5 older control-dizzy and 7 older control-not dizzy group members could not be included due to missing values for one or more of the control variables; ^b^ As VSS scores (i.e. dizziness severity) is a proxy for group membership between dizzy vs. non-dizzy controls, this variable was not entered; **NB2:** Step 1: Nagelkerke R^2^=.06; χ^2^(1)=9.85, p=0.002; AUC = .63, Specificity = .63, Sensitivity = .51, cut-off: .34; Step 2: Nagelkerke R^2^=.13; χ^2^(6)=23.45, p<0.001; AUC = .68, Specificity = .63, Sensitivity = .63, cut-off: .32; **Abbreviations:** Balance-VQ = Balance Vigilance Questionnaire; CI = Confidence interval; HADS = Hospital Anxiety and Depression Scale.

**Supplemental Material 5.** Linear regressions exploring the relationship between Balance-VQ and VSS-Total scores across the three groups (PPPD, ‘other’ vestibular disorder and older adult controls who reported experiencing dizziness in daily life).

| **MODEL 1: Relationship between Balance-VQ and VSS-Total in PPPD group (N=90)** | | | | | | |
| --- | --- | --- | --- | --- | --- | --- |
| Dependent variable: **Dizziness Severity (VSS-Total scores)** | | | | | | |
|  | *B* (*SE*) | | [95% CI] | *p* | *R*^2^ | *R*^2^ change |
| **Step 1** |  | |  |  | .18 (***p*<.001**) |  |
| *Intercept* | 3.59 (4.28) | | [-4.92, 12.10] | .404 |  |  |
| Balance-VQ | 0.83 (0.19) | | [0.46, 1.20] | **<.001** |  |  |
| **Step 2** |  | |  |  | .19 (***p*=.007**) | .01 **(***p*=.989**)** |
| *Intercept* | 6.87 (7.27) | | [-7.60, 21.34] | .348 |  |  |
| Balance-VQ | 0.83 (0.20) | | [0.43, 1.22] | **<.001** |  |  |
| Age (in years)  Gender *(reference – male)*  HADS-Anxiety | -0.04 (0.11)  -0.59 (2.79)  -0.04 (0.21) | | [-0.25, 0.17]  [-6.15, 4.96]  [-0.46, 0.38] | .689  .832  .862 |  |  |
| Depression Diagnosis *(reference = no)* | -0.88 (2.33) | | [-5.52, 3.75] | .705 |  |  |
| No. of medications > 4 *(reference = no)* | -0.80 (4.18) | | [-9.11, 7.51] | .849 |  |  |
| **MODEL 2: Relationship between Balance-VQ and VSS-Total in Vestibular group (N=84)** | | | | | | |
| Dependent variable: **Dizziness Severity (VSS-Total scores)** | | | | | | |
|  | *B* (*SE*) | | [95% CI] | *p* | *R*^2^ | *R*^2^ change |
| **Step 1** |  | |  |  | .25 **(*p*<.001)** |  |
| *Intercept* | -3.04 (3.30) | | [-9.60, 3.53] | .320 |  |  |
| Balance-VQ | 0.83 (0.16) | | [0.52, 1.15] | **<.001** |  |  |
| **Step 2** |  | |  |  | .38 (***p*<.001**) | .13 **(p=.010)** |
| *Intercept* | 9.76 (6.93) | | [-4.04, 23.57] | .163 |  |  |
| Balance-VQ | 0.54 (0.17) | | [0.20, 0.88] | **.002** |  |  |
| Age (in years)  Gender *(reference – male)*  HADS-Anxiety | -0.19 (0.07)  0.10 (3.17)  0.56 (0.25) | | [-0.33, -0.05]  [-6.22, 6.41]  [0.05, 1.06] | .**010**  .976  .**030** |  |  |
| Depression Diagnosis *(reference = no)* | 0.97 (1.94) | | [-2.89, 4.83] | .617 |  |  |
| No. of medications > 4 *(reference = no)* | 1.87 (2.51) | | [-3.13, 6.88] | .458 |  |  |
| **MODEL 3: Relationship between Balance-VQ and VSS-Total in *older adult* controls with dizziness (N=72)** | | | | | | |
| Dependent variable: **Dizziness Severity (VSS-Total scores)** | | | | | | |
|  | | *B* (*SE*) | [95% CI] | *p* | *R*^2^ | *R*^2^ change |
| **Step 1** | |  |  |  | .24 (***p*<.001**) |  |
| *Intercept* | | 0.35 (1.32) | [-2.29, 2.99] | .794 |  |  |
| Balance-VQ | | 0.39 (0.08) | [0.22, 0.55] | **<.001** |  |  |
| **Step 2** | |  |  |  | .30 (***p*<.001**) | .06 (*p*=.393) |
| *Intercept* | | 3.58 (5.69) | [-7.78, 14.94] | .532 |  |  |
| Balance-VQ | | 0.33 (0.10) | [0.14, 0.52] | **<.001** |  |  |
| Age (in years)  Gender *(reference – male)*  HADS-Anxiety | | -0.05 (0.08)  1.22 (1.20)  0.01 (0.15) | [-0.20, 0.10]  [-1.17, 3.62]  [-0.29, 0.30] | .473  .312  .964 |  |  |
| Depression Diagnosis *(reference = no)* | | 0.05 (1.16) | [-2.26, 2.36] | .966 |  |  |
| No. of medications > 4 *(reference = no)* | | 2.61 (1.29) | [0.04, 5.19] | .**047** |  |  |

**NB:** For each group, a few participants could not be included due to missing values for one or more of the control variables and/or VSS score (PPPD: N=7, Vestibular: N=13, Control: N=15). **Abbreviations:** Balance-VQ = Balance Vigilance Questionnaire; HADS-A = Anxiety subscale of the Hospital Anxiety and Depression Scale; VSS-Total = total score of the Vertigo Symptoms Scale.

**Supplemental Material 6.** ROC Curves and corresponding curve coordinates.


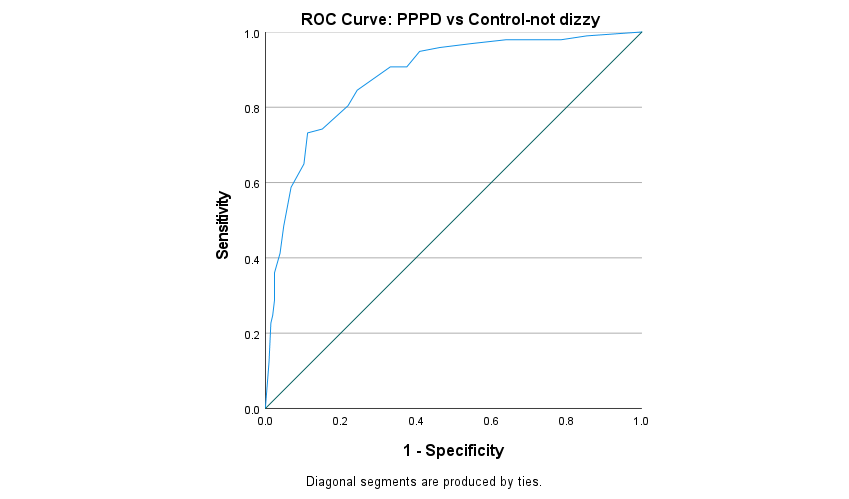

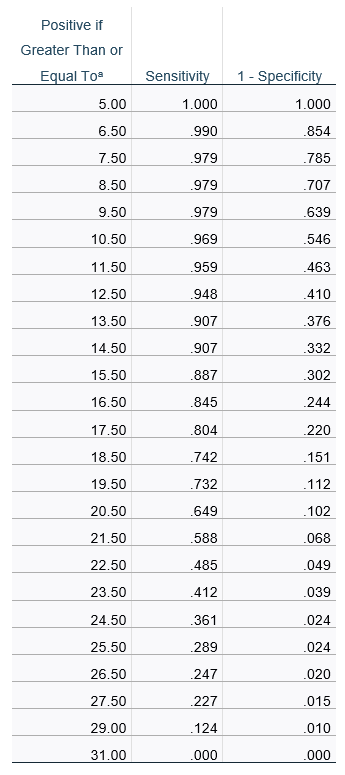


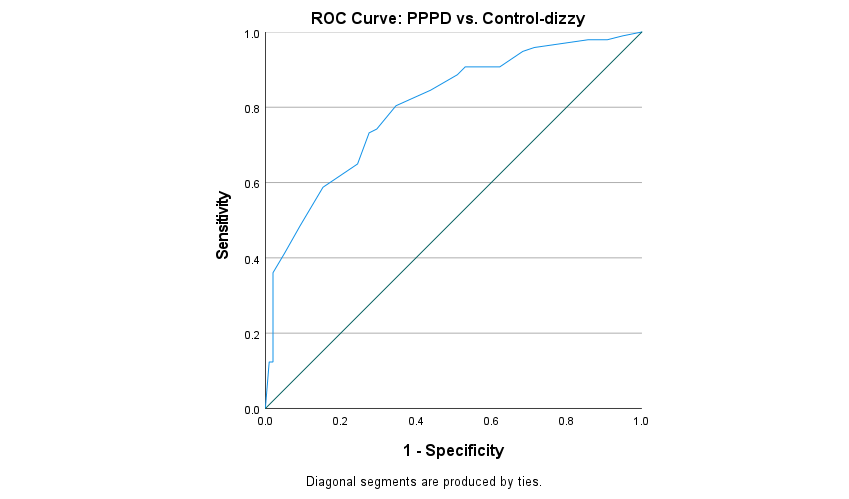

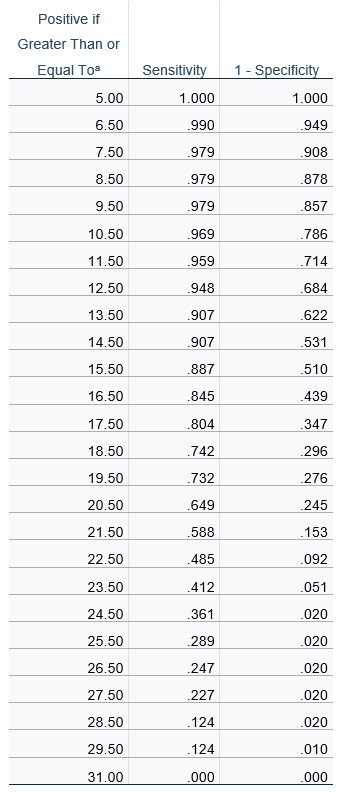


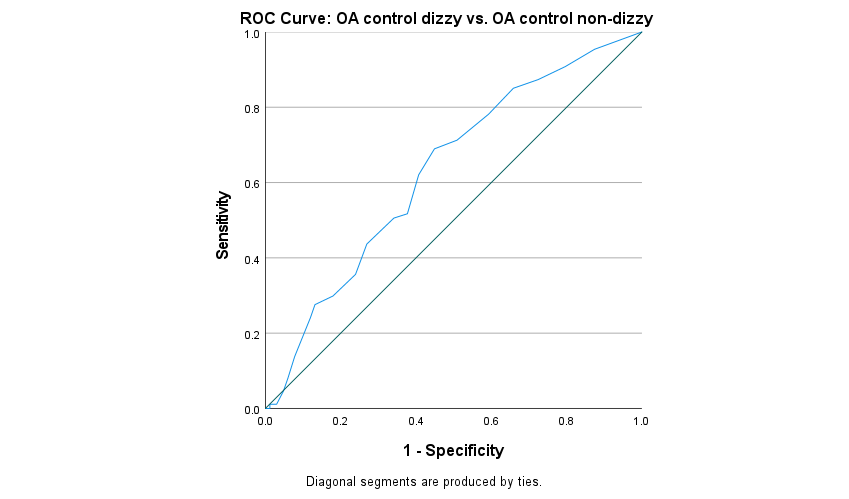

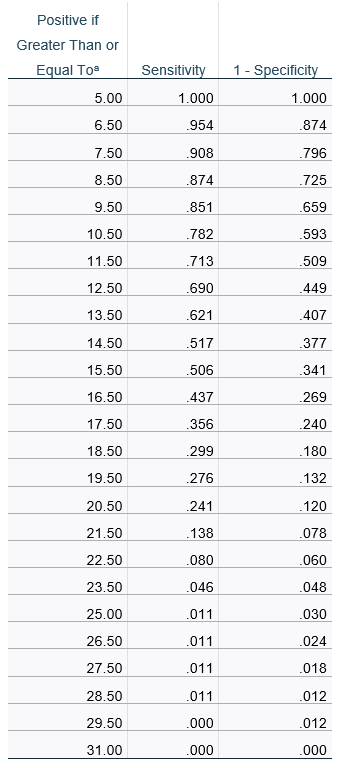


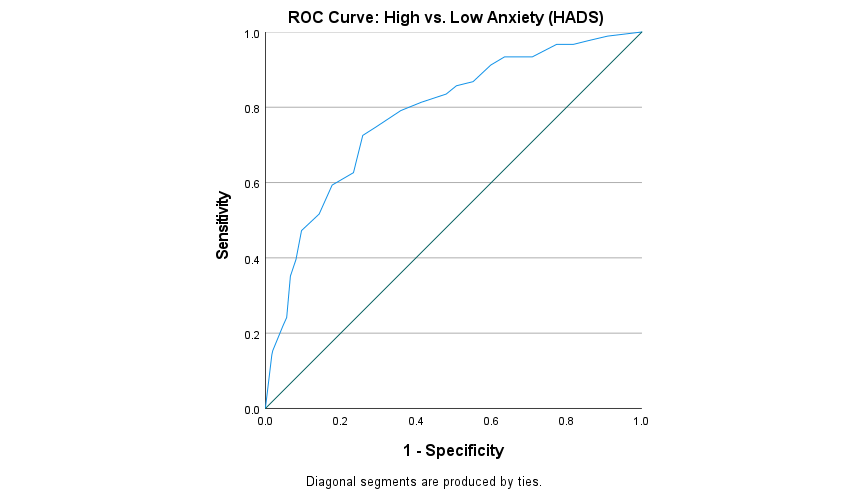

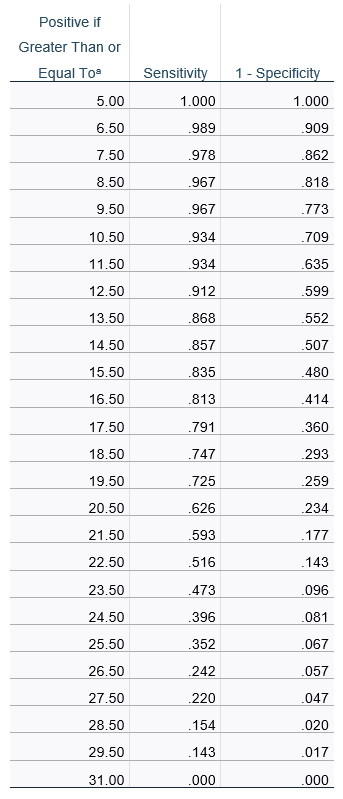


1. Hu L, Bentler PM. Cut-off criteria for fit indexes in covariance structure analysis: conventional criteria versus new alternatives. Struct Equ Modeling 1999; 6: 1–55. <https://doi.org/10.1080/10705519909540118> [↑](#footnote-ref-1)
2. Browne MW, Cudeck R. Alternative ways of assessing model fit. Sociol Method Res 1992; 21: 230–58. <https://doi.org/10.1177%2F0049124192021002005> [↑](#footnote-ref-2)
3. Chen FF. Sensitivity of goodness of fit indexes to lack of measurement invariance. Struct Equ Modeling 2007; 14: 464-504. <https://doi.org/10.1080/10705510701301834> [↑](#footnote-ref-3)
